# Health-Related Quality of Life in Colorectal Cancer Patients Treated With Liver Transplantation Compared to Chemotherapy

**DOI:** 10.3389/ti.2022.10404

**Published:** 2022-05-30

**Authors:** Tor Magnus Smedman, Tormod Kyrre Guren, Kjell Magne Tveit, Maria Thomsen, Marit Helen Andersen, Pål-Dag Line, Svein Dueland

**Affiliations:** ^1^ Oslo University Hospital, Oslo, Norway; ^2^ Institute of Clinical Medicine, University of Oslo, Oslo, Norway

**Keywords:** liver transplantation, quality of life, colorectal cancer, liver metastases, chemotherapy

## Abstract

Liver transplantation (LT) for patients with non-resectable colorectal liver metastases (CRLM) offers improved survival and has gained increased interest internationally the last years. The aim of this study was to describe the health-related quality of life (HRQoL) in patients with non-resectable CRLM receiving LT and how baseline HRQoL factors affect overall survival (OS). HRQoL data in the SECA (SEcondary CAncer) LT cohort was compared to data obtained from colorectal cancer patients starting first-line chemotherapy for metastatic disease in a clinical trial and data from a Norwegian normal population. HRQoL data from the QLQ-C30 questionnaire used in the SECA LT study and the NORDIC- VII study were reported. The relationship between patient-reported symptom burden at baseline and OS was investigated. In the SECA study longitudinal HRQoL assessment was used to describe the time until definitive deterioration as well as mean values at different time points. Patients in the SECA and NORDIC-VII studies reported similar baseline HRQoL. The median time until definitive deterioration in the transplanted patients was estimated to 36 months. In the SECA study appetite loss and pain at baseline had negative impact on OS (25.3 versus 71.7 months, *p* = 0.002 and 39.7 versus 71.7 months, *p* = 0.038, respectively). Despite a relapse in most of the LT patients the Global Health Score (GHS) remained good. Pain, and especially appetite loss at time of transplantation is associated with poor outcome after LT.

## GRAPHICAL ABSTRACT

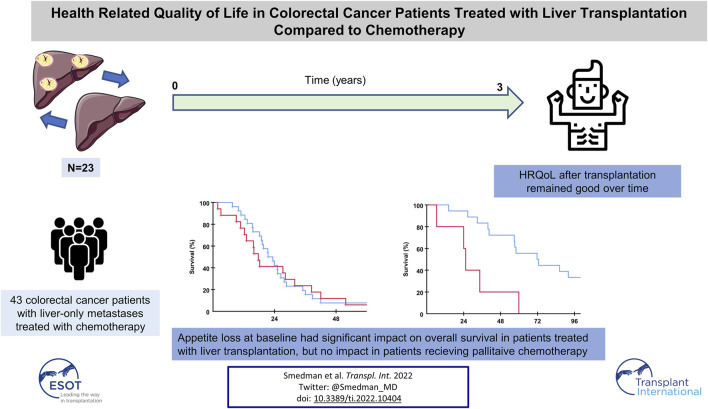

## Introduction

Colorectal cancer (CRC) is one of the most common malignancies worldwide. About 50% of CRC patients will develop distant metastases, with the liver as the most frequent site. Surgical resection is still considered as the only treatment with a curative potential, and 5-year overall survival (OS) after liver resection is reported to be 30%–50% [1]. Only about 20% of patients with metastatic disease are candidates for liver resection, and the majority of are treated with palliative chemotherapy with a median OS of approximately 2 years, and a 5-year OS of about 10% [2, 3].

Liver transplantation (LT) has been investigated as a treatment option in patients with non-resectable colorectal liver metastases (CRLM), and published results indicate that selected patients may obtain long-term survival following LT [4-7]. We have previously reported the outcome of the SECA (SEcondary CAncer) study, in which 21 patients with unresectable CRLM underwent LT, with a 5-year OS rate of 60% [6]. Several other transplant centers have ongoing clinical trials for incorporating LT as treatment of unresectable CRLM; France (TRANSMET, NCT02597348), Canada (Toronto Protocol, NCT02864485), Germany (LIVERT (W) OHEAL, NCT03488953), Italy (COLT, NCT03803436), and Sweden (SOULMATE, NCT04161092).

Quality of life (QoL) is increasingly recognized as an important measure of outcome after solid organ transplantation as well as during palliative chemotherapy for metastatic disease [8-11]. Health-related quality of life (HRQoL) has been shown to be a significant predictor of treatment outcome in cancer patients [11-13]. In metastatic CRC, baseline physical function (PF) and global QoL score were independently associated with OS [14, 15].

CRC patients treated with liver resection have an overall deterioration in HRQoL after surgery followed by a rise in HRQoL scores to baseline after 3–6 months. This transient fall in HRQoL values has also been reported in CRC patients who underwent LT as well as CRC patients with peritoneal metastases treated with cytoreductive surgery and intraperitoneal chemotherapy [16, 17].

Efficient, reliable, and clinically meaningful HRQoL assessment and interpretation have their limitations. Due to its longitudinal nature as well as missing data and patient drop-outs, identification of clinical meaningful appropriate time points and interpretation of results in HRQoL studies can be challenging [18]. Cancer patients have been found to report HRQoL levels that are similar to those of healthy people, even if their objective clinical health status deteriorates significantly [19, 20]. This process of accommodating to the illness is referred to as the “response shift,” and may further complicate HRQoL data interpretation [21]. Time until definitive deterioration (TUDD) in the QoL score has been established as a method of longitudinal analysis in the field of oncology [22-24], and allows data to be analyzed even if some questionnaires are missing, and produces meaningful results such as Kaplan-Meier curves that are easily interpreted by clinicians.

Patients included in clinical trials differ from other patients since they are selected based on various clinical parameters, and thus patients included in trials obtain longer OS despite receiving the same treatment as patients outside of trials [25]. A good performance state is often a criterion for inclusion, and most patients included in CRC clinical trials have an Eastern Cooperative Oncology Group (ECOG) performance status of 0 or 1.

It is of interest to establish whether the long OS obtained in the SECA LT study may have been influenced by selection of patients with superior QoL compared to patients in other CRC cancer trials. We sought to investigate this by using similar patients receiving first-line standard chemotherapy in the NORDIC-VII study [26] as a reference group.

There is limited knowledge about HRQoL of mCRC patients treated with LT [16, 27]. Surviving patients in the SECA study have a median follow-up of 11 years. The aim of this manuscript was to report updated OS numbers and give a more in-depth HRQoL analysis with TUDD and compare the results to reference HRQoL data from a healthy population. Furthermore, we investigated whether baseline HRQoL parameters influencing OS in the SECA study also had similar effects in patients receiving palliative first-line chemotherapy in the NORDIC-VII study. Lastly, we investigated whether recurrent disease after LT had an impact on patient-reported HRQoL.

## Materials and Methods

### Patients

The SECA study (NCT01311453) was a prospective pilot study which included 23 patients that received LT as treatment for non-resectable liver-only CRLM [6]. The patients had received one to three lines of chemotherapy prior to inclusion (Supplementary Table S1). The NORDIC-VII study (NCT 00145314) was a randomized, multicenter, three-arm phase III clinical trial that randomized patients 1:1:1 to Nordic FLOX (arm A), FLOX + cetuximab (arm B), and FLOX intermittently + cetuximab continuously (arm C) arms [26]. The Nordic FLOX regimen was administered every 2 weeks as 85 mg/m^2^ of oxaliplatin over 1 h (30–90 min) on day 1, 500 mg/m^2^ of fluorouracil as a bolus infusion (<5 min), followed 30 min later by 60 mg/m^2^ of folinic acid as a bolus infusion (<10 min) on days 1 and 2.

**FIGURE 1:**
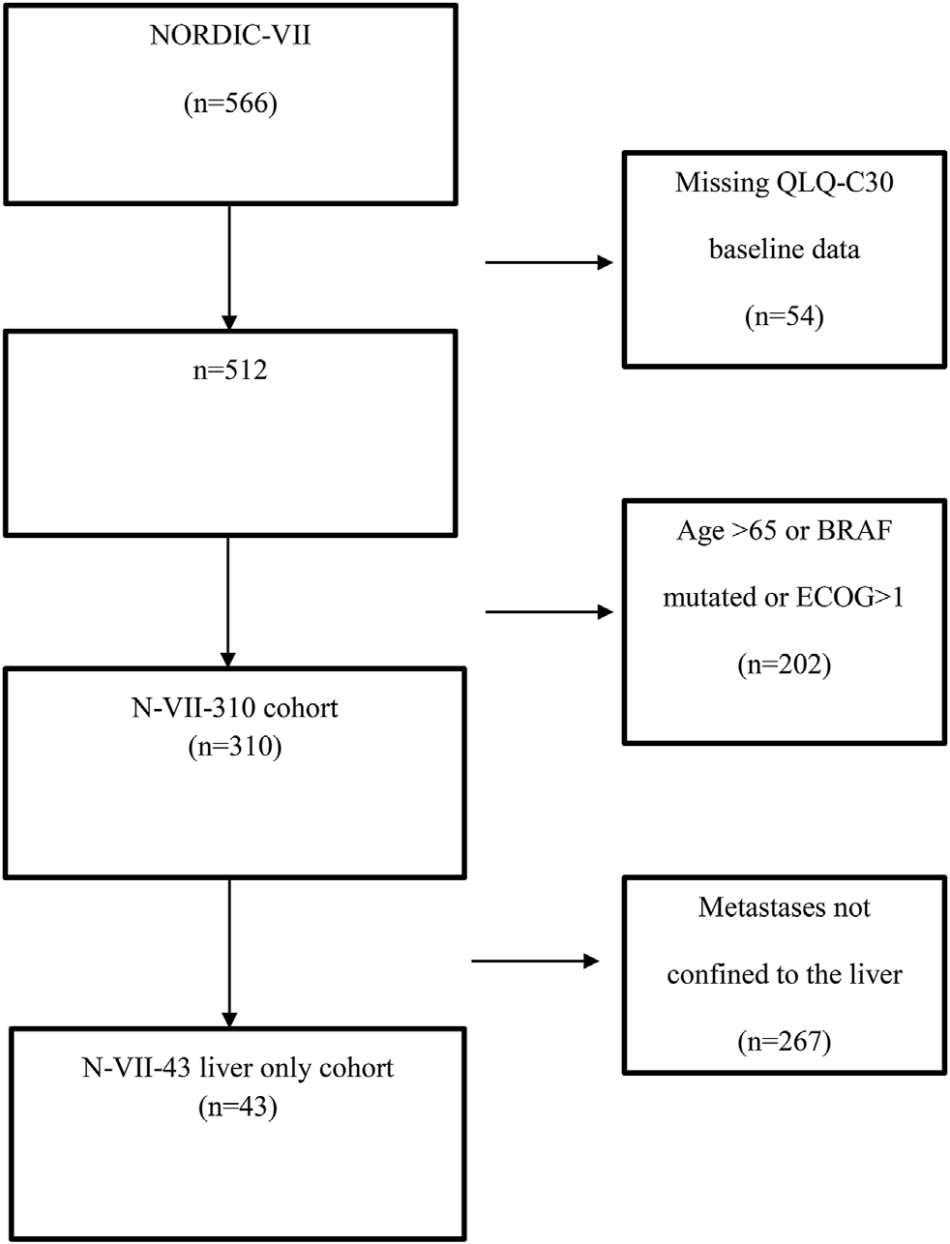
Flow chart showing the selection of NORDIC-VII patients.

The study included 566 patients, 512 patients had completed QLQ-C30 before the start of treatment, and 310 of these 512 patients had an ECOG status of 0–1, age ≤65 years, and BRAF wild-type, labeled as N-VII-310 (Figure 1). A total of 43 of these 310 patients had metastases confined to the liver, labeled as N-VII-43 (Figure 1). These selection criteria were chosen with the intent to align a cohort of patients with similar characteristics as the transplanted patients in the SECA study, as we have previously reported [4].

The SECA study and the NORDIC-VII study had received approval from the Regional Ethics Committee and Institutional Review Board. All patients had signed informed consent before inclusion.

### Health-Related Quality of Life Assessment

HRQoL was assessed using the European Organization for Research and Treatment of Cancer Quality of Life Questionnaire Core 30 (QLQ-C30) [28]. The QLQ-C30 is a 30-item cancer-specific self-report questionnaire that generates five functioning scales, one overall QoL scale, three symptom scales, and six single items. Scoring was performed according to the EORTC scoring manual. Symptoms are scored on a categorical scale ranging from 1 (not at all) to 4 (very much), whereas the two items assessing overall health and QoL are scored from 1 to 7 and constitute the global health status (GHS). For the five function scales and the GHS, a higher score indicates a better level of functioning, whereas for the symptom scales and items, a high score indicates a higher level of symptomatology. The responses were linearly transformed to range from 0 to 100.

Patient-reported HRQoL in the SECA study was obtained at baseline (before LT), after 3 months and 6 months, then every 6 months for up to 3 years. In the NORDIC-VII study, assessment was obtained at baseline (before the start of treatment) and then at every four cycles (approximately every 2 months) until progressive disease (PD) or end of treatment. In the SECA study, all patients were reminded by phone if the questionnaire was not received.

### Statistical Methods

Survival data were estimated using the Kaplan-Meier method and outcomes between groups were compared using the log rank test. For comparison between two groups with categorical variables, the two-sided Fisher’s exact test was used. To comparing mean values of HRQoL, the Mann-Whitney U test was used. For all tests, a two-sided *p <* 0.05 was considered statistically significant. Statistical analyses were performed using SPSS® version 25 (IBM, Armonk, New York, USA). The cut-off for survival analysis was 1 August 2019 for the SECA study and 20 April 2014 for the NORDIC-VII study.

### Analysis of Time Until Definitive Deterioration of Health-Related Quality of Life

A change in HRQoL score of 5–10 points or more on the 0–100 scale is generally considered clinically significant, and a cut-off of 10 points has previously been used in HRQoL analysis [29-31]. However, in the TUDD method, the cut-off is frequently set at ≥ 5 points [22-24]. In this report, TUDD was defined as the interval between baseline and the first decrease in HRQoL score ≥5 points compared to baseline without any further improvement of ≥5 points or any further available data or death, as described by Bonnetain et al. [23]. Surviving patients were censored at the last follow-up if a ≥5 decrease from baseline was not observed, or if a ≥5 point reduction in HRQoL in which a secondary >5 point improvement in HRQoL score within 5 points from baseline was observed.

The differences between mean HRQoL scores at baseline and mean scores obtained at 6 months were investigated in the different cohorts. The 6-month time point was chosen since both studies had collected questionnaires 6 months after the start of treatment. Furthermore, previous reports in the SECA study have shown that patients had a fall in HRQoL at 3 months after LT, followed by a rise to baseline at 6 months.

## Results

Key clinical parameters in the three study cohorts are given in Table 1. The cohorts were relatively similar with median ages of 54–58 years, 70%–74% had ECOG performance statuses of 0, and KRAS was mutated in 26%–39% of the patients.

**TABLE 1:** Baseline characteristics of the three study cohorts.

	SECA *n* = 23	N-VII-43 *n* = 43	N-VII-310 *n* = 310
Liver-only metastases	Yes	Yes	No
Primary tumor removed	Yes	Yes	Not required
BRAF mutation	No	No	No
PET	Yes	Not required	Not required
Prior chemotherapy	Yes	No	No
Age ≤65 years	Yes	Yes	Yes
Age, median (range), y	54 (44–64)	58 (43–65)	58 (26–65)
Sex (male/female)	57% male	72% male	58% male
Site	57% colon	63% colon	52% colon
ECOG 0	74%	70%	70%
CEA, median (range), µg/L	9 (1–2002)	40 (0.5–8260)	N/A
ALP > UNL	48%	67%	49%
KRAS mutation	35%	26%	39%
CRP, median (range), mg/L	7 (1–256)	19 (0–250)	14 (0–766)

SECA, SEcondary CAncer study; N-VII-43, NORDIC-VII liver-only cohort, 43 patients; N-VII-310, NORDIC-VII cohort (including patients with extra-hepatic metastatic disease), 310 patients; PET, photon emission tomography; ECOG, eastern cooperation oncology group; CEA, carcinoembryonic antigen; ALP, alkaline phosphatase; KRAS; kirsten rat sarkoma virus; CRP, C-reactive protein.

### Response to the QLQ-C30 Questionnaire

In the SECA cohort, the rate of patients responding to the questionnaire at 6 months was 21 of 23 patients (91%) as compared to 16 of 31 patients (52%) in the N-VII-43 cohort and 88 of 211 patients (42%) in the N-VII-310 cohort when excluding patients with progression-free survival (PFS) less than 6 months (patients in the NORDIC-VII study did not receive a questionnaire after disease progression). The OS in patients not responding to the questionnaire at 6 months was similar to those who responded in both NORDIC-VII study cohorts (23.4 months and 25.2 months, *p* = 0.908 in N-VII-43 and 25.3 months and 27.3 months, *p* = 0.428 in N-VII-310). In the SECA cohort, the two patients that did not answer the questionnaire at 6 months had an OS of 6.4 months and 32.8 months, respectively, which was significantly shorter compared to the patients that answered the questionnaire (median OS 59.9 months, *p* = 0.003).

### Baseline Mean Values

Patients in the SECA cohort had similar baseline mean HRQoL scores compared to both the N-VII-43 (liver only) and total N-VII-310 cohorts (Table 2). The HRQoL values were similar in the N-VII-43 and N-VII-310 cohorts. In the SECA cohort, baseline HRQoL mean values were not statistically significantly different for patients who had received one line of chemotherapy compared to those who had received two or three lines of chemotherapy prior to LT (Supplementary Table S2).

**TABLE 2:** Health-related quality of life mean values at baseline and at 6 months in the three study cohorts.

	SECA HRQoL mean values at baseline (95% CI interval) (*n* = 23)	SECA HRQoL mean values at 6 months (95% CI interval) (*n* = 21)	N-VII-43 HRQoL mean values at baseline (95% CI interval) (*n* = 43)	N-VII-43 HRQoL mean values at 6 months (95% CI interval) (*n* = 16)	N-VII-310 HRQoL mean values at baseline (95% CI interval) (*n* = 310)	N-VII-310 HRQoL mean values at 6 months (95% CI interval) (*n* = 99)
Physical functioning	89.2 (83.4–95.0)	85.7 (78.5–93.0)	85.6 (79.7–91.5)	83.3 (75.0–91.6)	85.3 (83.4–87.2)	82.4 (79.4–86.0)
Social functioning	71.0 (58.7–83.3)	70.6 (57.7–83.5)	83.3 (76.9–89.8)	83.3 (75.0–91.6)	80.4 (77.7–83.1)	79.8 (75.3–84.0)
Role functioning	81.9 (72.2–91.6)	73.8 (60.8–86.9)	69.0 (58.279.7)	65.6 (52.1–79.2)	70.3 (66.7–73.8)	69.0 (63.0–74.4)
Emotional functioning	81.9 (74.0–89.8)	83.7 (74.2–93.2)	80.3 (74.5–86.0)	85.4 (77.4–93.8)	75.9 (73.6–78.1)	87.0 (83.2–90.4)
Cognitive functioning	87.7 (79.8–95.6)	88.9 (79.5–98.3)	94.6 (91.1–98.1)	88.5 (80.8–96.3)	89.7 (87.9–91.6)	89.7 (87.6–93.5)
Global QOL	77.2 (69.9–84.5)	80.2 (72.2–88.2)	69.8 (62.5–77.1)	66.1 (57.8–74.5)	69.0 (66.5–71.4)	70.2 (66.3–74.0)
Fatigue	27.1 (18.8–35.4)	24.9 (15.2–34.6)	29.2 (22.1–36.3)	36.1 (25.0–47.3)	31.1 (28.5–33.6)	31.1 (26.7–35.3)
Nausea/vomiting	4.3 (0.5–8.2)	5.6 (1.2–9.9)	6.2 (2.2–10.2)	2.1 (−0.95–5.1)	6.8 (5.2–8.4)	4.9 (3.0–6.8)
Pain	11.6 (3.6–19.6)	20.6 (11.1–30.2)	16.7 (9.8–23.6)	5.2 (−1.8–12.3)	21.0 (18.3–23.7)	12.2 (8.8–16.2)
Dyspnea	7.2 (1.2–13.3)	19.0 (10.0–28.1)	11.6 (4.6–18.7)	14.6 (3.4–25.8)	14.4 (11.9–16.9)	18.5 (14.6–23.1)
Sleeping disturbances	26.1 (13.1–39.1)	33.4 (21.6–45.1)	18.6 (12.2–25.1)	12.5 (1.5–23.5)	26.1 (23.2–29.1)	13.4 (9.1–17.8)
Appetite loss	8.7 (0.9–16.5)	14.3 (4.0–24.5)	18.6 (9.8–27.4)	6.3 (−0.9–13–4)	19.9 (16.7–23.1)	10.5 (6.5–13.7)
Constipation	13.0 (3.6-22-5)	7.9 (−1.5–17.4)	12.4 (5.7–19.2)	12.5 (3.6–21.4)	14.0 (11.3–16.6)	8.5 (4.9–11.9)
Diarrhea	14.5 (7.2–21.8)	26.9 (14.6–39.3)	15.1 (7.4–22.8)	8.3 (−1.9–18.6)	15.3 (12.6–17.9)	11.9 (7.5–16.0)
Financial impact	17.4 (3.1–31.7)	14.3 (2.0–26.6)	13.2 (4.8–21.6)	16.7 (3.7–29.6)	10.6 (8.1–13.2)	14.0 (8.1–18.9)

SECA, SEcondary CAncer study; CI, confidence interval; N-VII-43, NORDIC-VII liver-only cohort, 43 patients; N-VII-310, NORDIC-VII cohort (including patients with extra-hepatic metastatic disease), 310 patients; QOL, quality of life.

Patients with PD within 6 months in either of the NORDIC-VII cohorts did not have significantly different baseline mean values for GHS, PF, appetite loss fatigue, pain, or diarrhea (Supplementary Table S3).

### Baseline Mean Values Compared to Mean Values at 6 months

As previously shown, there was a statistically significant higher mean value of dyspnea at 6 months compared to baseline in the SECA cohort [16], with scores of 19 and 7.2, respectively, *p* = 0.032. No other HRQoL parameters were statistically significantly different. However, the mean value for diarrhea increased by more than 10 points at 6 months compared to baseline, but this increase was not statistically significant (*p* = 0.131, Table 2). In comparison, patients in the N-VII cohorts had statistically significantly less pain 6 months after starting palliative chemotherapy (5.2 versus 16.7, *p* = 0.027 for N-VII-43 and 12.2 versus 21.0, *p* < 0.001 for N-VII-310) and none of the patient-reported symptom scores were significantly higher.

### Baseline Values and Influence on Survival

**FIGURE 2:**
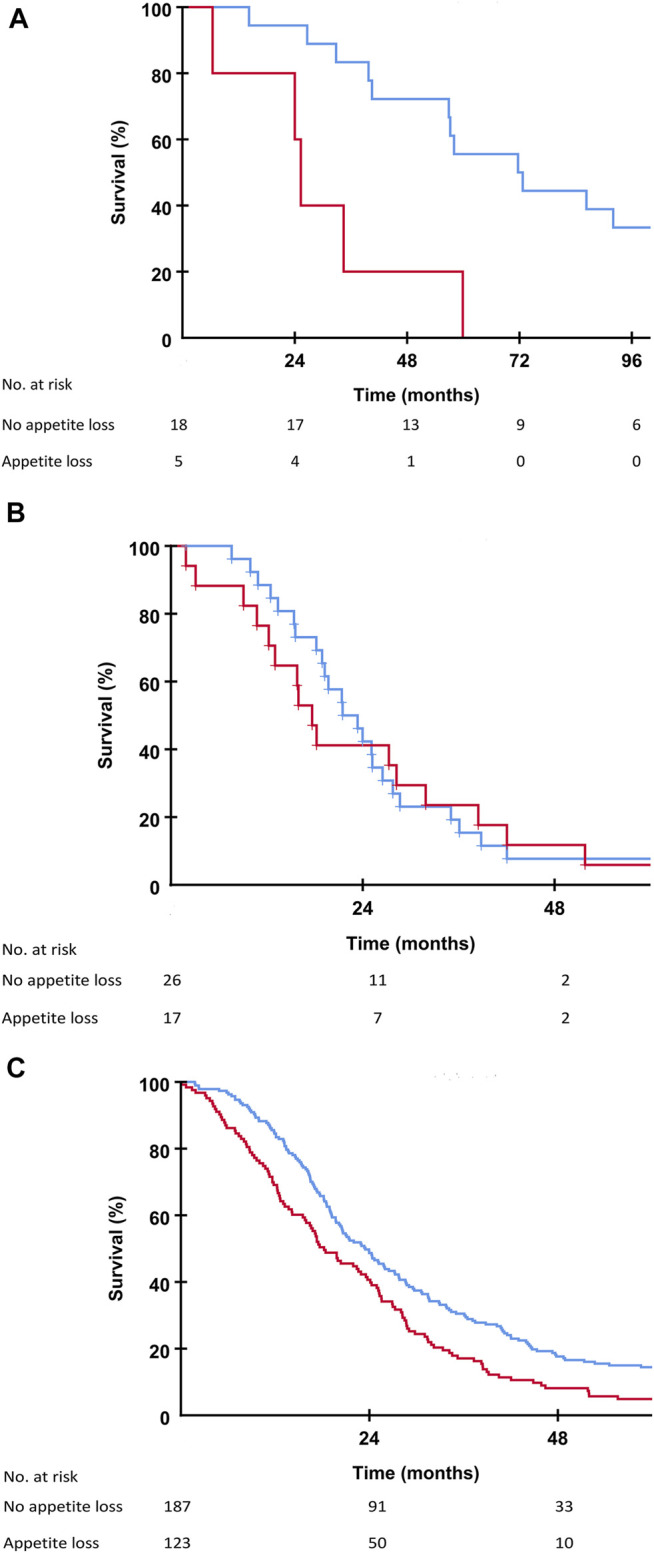
**(A)** Kaplan-Meier plot showing overall survival in the SECA study in patients with no appetite loss (blue line) and patients with appetite loss (red line) at the time of liver transplantation. Difference between the two groups was *p* = 0.002. **(B)** Kaplan-Meier plot showing overall survival in the N-VII-43 liver-only cohort in patients with no appetite loss (blue line) and patients with appetite loss (red line) at the start of chemotherapy. Difference between the two groups was *p* = 0.658. **(C)** Kaplan-Meier plot showing overall survival in the N-VII-310 cohort in patients with no appetite loss (blue line) and patients with appetite loss (red line) at the start of chemotherapy. Difference between the two groups was *p* = 0.001.

**FIGURE 3:**
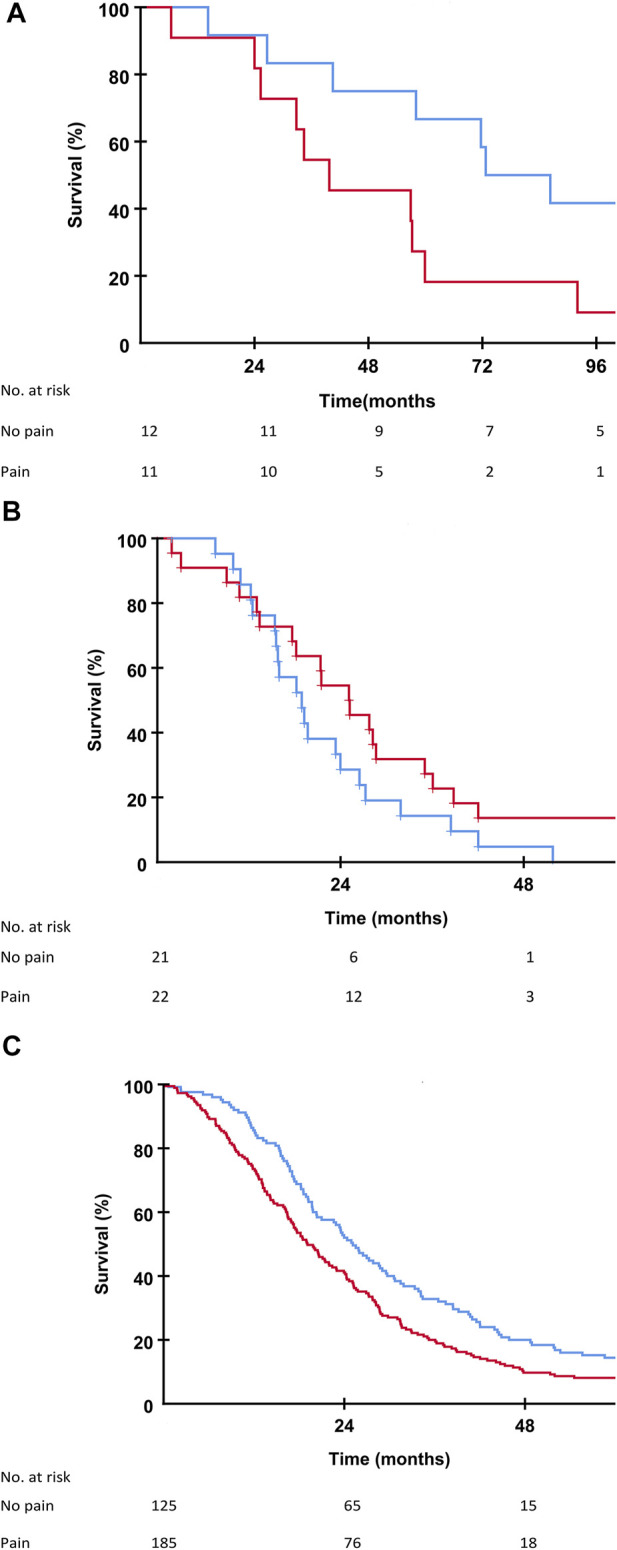
**(A)** Kaplan-Meier plot showing overall survival in the SECA study in patients with no pain (blue line) and patients with pain (red line) at the time of liver transplantation. Difference between the two groups was *p* = 0.038. **(B)** Kaplan-Meier plot showing overall survival in the N-VII-43 liver-only cohort in patients with no pain (blue line) and patients with pain (red line) at the start of chemotherapy. Difference between the two groups was *p* = 0.129. **(C)** Kaplan-Meier plot showing overall survival in the N-VII-310 cohort in patients with no pain (blue line) and patients with pain (red line) at the start of chemotherapy. Difference between the two groups was *p* = 0.002.

Patients in the SECA, N-VII-43, and N-VII-310 cohorts were followed-up for more than 5 years. Surviving patients in the SECA study had a median follow-up of 11 years (range 8.4–12.6 years). In the SECA cohort, patients with appetite loss at baseline (*n* = 5) had a significantly shorter OS (median OS 25.3 months) compared to those with no appetite loss (*n* = 18) who had a median OS of 71.7 months (*p* = 0.002, Figure 2A). The Kaplan-Meier-calculated 5-year OS rate in patients with appetite loss was 0% compared to 56% in patients with no appetite loss at baseline. Patients with appetite loss did not have significantly different values for tumor size, number of metastases, CEA, or positron emission topography metabolic tumor volume (PET-MTV, data not shown), however, these had a significantly poorer mean baseline GHS of 60 as compared to patients without appetite loss who had a mean GHS of 82 (*p* = 0.023). Patients with CRP level ≥10 mg/L at the time of LT had a significantly reduced OS of 39.8 months compared to patients with CRP <10 mg/L who had an OS of 59.9 months (*p* = 0.046). A reduced OS was also observed in SECA patients who reported pain at baseline; these patients (*n* = 11) had a significantly shorter median OS of 39.7 months compared to patients without pain (*n* = 12) who had a median OS of 72.7 months (*p* = 0.038, Figure 3A). The Kaplan-Meier-calculated 5-year OS for patients with pain versus no pain was 18.2% and 66.7%, respectively.

Patients in the SECA cohort with fatigue >30 at baseline (*n* = 12) had a median survival of 34.4 months compared to patients with fatigue ≤30 (*n* = 11) who had a median OS of 72.7 months, this difference, however, was not statistically significant (*p* = 0.183). The Kaplan-Meier-estimated 5-year OS for patients with fatigue versus no fatigue was 33.4% and 54.5%, respectively.

In the N-VII-43 cohort, there was no statistically significant impact on OS regarding appetite loss, pain, or fatigue at baseline (Figures 2B, 3B).

In the N-VII-310 cohort, however, for all these symptom variables there was a relationship between impaired OS for patients with appetite loss compared to those without appetite loss (18.2 months versus 23.5 months, *p* = 0.001, Figure 2C). Median OS for patients who reported pain at baseline was 19.1 months versus 25.1 months for patients with no pain (*p* = 0.002, Figure 3C). Patients with fatigue >30 had a median OS of 20.2 months compared to 28.5 months in patients with fatigue ≤30 (*p* = 0.001).

### Quality of Life After Relapse

A total of 16 of 23 patients were alive at the time of last HRQoL data collection (36 months) after LT, and 14 of these 16 patients had relapse within 36 months. Mean GHS for the 14 patients with relapse was 79.6 at 36 months as compared to 70.2 at baseline (*p* = 0.281). For all patients with relapse within 36 months (*n* = 21) after LT, the difference of GHS mean values at the first time point right after relapse compared to prior to relapse was not clinically relevant or statistically significant (62.3 as compared to 70.2 prior to relapse, *p* = 0.330).

Patients with Clavien-Dindo [32] ≥grade IIIA post-operative complications (*n* = 12) had a fall in GHS from baseline (75.7) to 3 months after LT (62.9), but then a rise to baseline values at 6 months after LT (78.8). Patients with Clavien-Dindo < IIIA complications (n = 11) had similar values at baseline, 3 months, and 6 months of 78.8, 64.4, and 81.6, respectively. There was no statistically significant difference between patients with Clavien-Dindo ≥ grade IIIA and grade < IIIA at either of these time points (*p* = 0.702, *p* = 0.92, *p* = 0.451).

### Longitudinal Quality of Life Analysis

**FIGURE 4:**
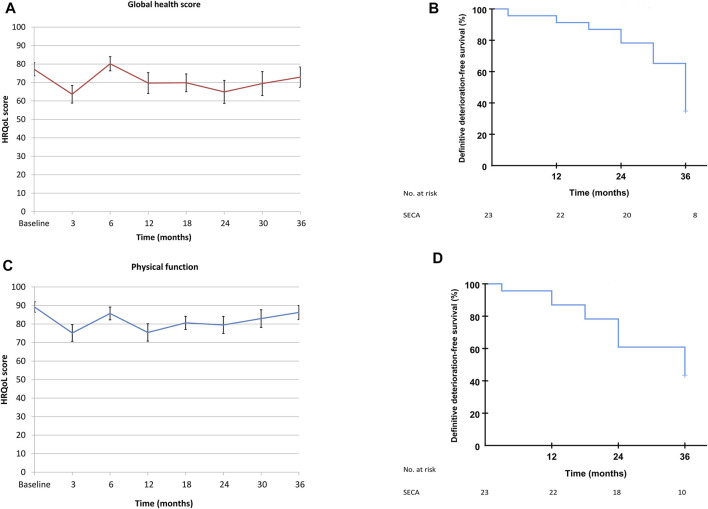
**(A)** SECA mean GHS values, from baseline to end of follow-up at 3 years. Data are from Figure 1 in Dueland et al., BJS Open, 2019 [16]. **(B)** Time until definitive deterioration of GHS in the SECA study. **(C)** SECA mean PF values, from baseline to end of follow-up at 3 years. Data are from Figure 1 in Dueland et al., BJS Open, 2019 [16]. **(D)** Time until definitive deterioration of PF in the SECA study.

The mean values in the SECA cohort of GHS and PF had a clinically important and statistically significant decrease of more than 10 points at 3 months post-LT [16] with values of 77.2–63.6, *p* = 0.034 for GHS and 89.2–75.2, *p* = 0.007 for PF, but the patient-reported HRQoL increased within 10 points back to baseline at 6 months without a further clinically relevant decrease until the end of HRQoL follow-up at 3 years (Figures 4A,C).

Median TUDD for both GHS and PF was 36 months in the SECA cohort (95% confidence interval 33 months–39 months for GHS and 22–50 months for PF, Figures 4B,D).

### Control Population Data

Control population HRQoL data were collected from a randomly selected sample of 3000 people from the Norwegian population as published by Hjermstad et al. [33]. The data were adjusted for age and sex according to the SECA population (Table 3). Compared to the control population, the SECA patients had clinically relevant higher values for diarrhea starting at 3 months post-LT, and the values remained high at all the following time points, with a slight drop at 3 years after LT. Furthermore, the SECA patients had higher scores for sleep disturbance and appetite loss at 6 months post-LT, but these parameters decreased to similar values as the control population within 3 years post-LT.

**TABLE 3:** Age and sex-adjusted SECA mean values compared to the control population.

	Control population^a^ *n* = 1965	SECA baseline *n* = 23	SECA 6 months *n* = 21	SECA 3 years *n* = 16
Physical functioning	87.7	89.2	85.7	86.3
Social functioning	84.9	71.0	70.6	75.0
Role functioning	93.5	81.9	73.8	73.0
Emotional functioning	83.5	81.9	83.7	84.9
Cognitive functioning	87.3	87.7	88.9	88.6
Global QOL	73.7	77.2	80.2	73.0
Fatigue	27.9	27.1	24.9	28.5
Nausea/vomiting	3.4	4.3	5.6	1.0
Pain	23.6	11.6	20.6	16.7
Dyspnea	14.2	7.2	19.0	12.5
Sleeping disturbances	22.2	26.1	33.4	20.8
Appetite loss	4.4	8.7	14.3	4.2
Constipation	10.6	13.0	7.9	16.7
Diarrhea	10.9	14.5	26.9	20.8
Financial impact	10.9	17.4	14.3	8.4

a Adjusted for age and sex.

SECA, SEcondary CAncer study; QOL, quality of life.

## Discussion

Over the last 20–30 years, surgical technique and post-operative care following LT have improved significantly, and, in addition to the development of more effective immunosuppression regimens, have led to dramatic progress in patient survival and post-LT morbidity rates [34, 35]. Despite these improvements, LT is a major surgical intervention with a number of potential complications, and major postoperative complications have been described in transplanted CRC patients [6]. Regardless of the rate of complications, LT patients still have a long post-operative hospitalization period. HRQoL, therefore, as one may expect, decreases in the first 3 months after LT, but increases to baseline levels around 6 months post-LT [16].

Based on encouraging results and the number of ongoing prospective trials throughout the world examining LT in CRC patients, LT for non-resectable CRLM may in the future become an established part of the treatment algorithm for selected patients with an otherwise dismal prognosis. In addition to OS and DFS, it is of importance to assess the impact of LT on HRQoL in these patients.

Both pain and appetite loss at baseline had a significant impact on OS in the SECA study. Patients without appetite loss at baseline in the SECA study had a considerable survival benefit of almost 4 years compared to transplanted patients with appetite loss, who had a survival rate comparable to the NORDIC-VII cohorts (Figures 2A–C). The SECA patients without appetite loss at baseline had a median OS of almost 6 years. Three of the five patients with appetite loss at baseline in the SECA cohort were on the last line of chemotherapy and thus had an anticipated OS of approximately 6 months [36]. However, appetite loss was independent of tumor load at the time of LT.

Increased CRP at the time of LT also seemed to predict poorer survival, independently of appetite loss. Similarly to the present findings, Thomsen et al. showed that increased CRP at the start of chemotherapy was associated with impaired OS in the NORDIC-VII study [37].

Baseline HRQoL has been shown to be associated with survival postoperatively in the setting of hepatic resection for both primary and secondary liver malignancies [38, 39]. As reported by Rees et al., weight loss, abdominal pain, altered taste, fatigue, and problems with sex life were all factors associated with poorer survival in patients treated with liver resection for CRC liver metastases [38]. However, appetite loss did not impact survival in these patients. Appetite loss, pain, and fatigue did not impact OS significantly in the 43 patients with liver-only metastases in the NORDIC-VII study, but this may be explained by the relatively low number of patients as there was a significant difference in the N-VII-310 cohort which included patients with extra-hepatic metastases. Inferior OS in mCRC patients with a high symptom burden at baseline has been reported previously. Maisey et al. showed that patients with few symptoms had a survival benefit of up to 10 months [15] and pain at baseline was also a negative prognostic factor in mCRC patients [40].

The patients in the SECA study had all been treated with chemotherapy prior to inclusion, some had received up to three lines of chemotherapy and received treatment over several years and some patients even had progressive disease at the last line of chemotherapy prior to LT. The patients in the NORDIC-VII study were at the time of inclusion newly diagnosed with metastatic disease and untreated. Despite this, baseline HRQoL values at the time of inclusion were not significantly different and patients with liver-only metastases in the NORDIC VII-population did not have different baseline HRQoL compared to patients with metastases in other organs or multiple sites. This suggests that SECA patients had similar HRQoL to newly diagnosed patients starting palliative chemotherapy.

The scarcity of donor organs for LT is a major challenge in all countries. In the USA, the waiting list mortality rate of about 20% is driven primarily by low organ availability [41]. The selection of patients for LT with the potential for long OS is therefore of major importance. Several clinical and pathological factors seem to be important for patient selection; tumor size <5.5 cm, CEA levels at time of LT < 80 μg/L, time from CRC surgery to LT more than 2 years, and stable disease or partial response to chemotherapy at the time of LT have all been independently associated with better outcome [6]. The results in this report indicate that appetite and pain at baseline could also be factors to aid in selecting patients for LT, although the subjective nature of these parameters is challenging. If these criteria were more objective, it is clear that patients with pain and, in particular, loss of appetite at baseline, should not be considered for LT.

Two different approaches were used to evaluate the longitudinal GHS and PF in the SECA cohort; the time until definitive deterioration with a five point cut-off and the mean HRQoL values at the different time points up to 3 years following LT (Figures 4A–D). In the latter method, we showed that there was a clinically relevant decrease in mean values of GHS and PF at 3 months post-LT, followed by an increase to baseline from 6 months and onward. When the TUDD method was used in the same SECA dataset, however, a gradual deterioration rate following LT was observed with a median TUDD of 36 months for both GHS and PF. Figures 4A–D illustrate how the same longitudinal HRQoL data can be visualized differently. The TUDD method calculates the rate of patients with definitive deterioration at different time points, and does not take into account the patients that in fact have improved HRQoL scores over time. The mean values, naturally, include both the patients with a decline and increase in values over time, but do not compensate for missing data, and this method reflects only patients alive and willing or capable of answering the questionnaires. However, the response rate of patients alive in the SECA-study at all time points was >90%. In the N-VII study cohorts, there were relatively stable mean values from baseline to 6 months, which is in concordance to what has previously been reported by Thomsen et al. where they showed that values for GHS, PF, fatigue, and pain were stable from the start of treatment to 6 months in patients in the whole NORDIC-VII study [42]. However, due to the low rate of responders at 6 months in the NORDIC-VII study, it is likely that the majority of patients with increased symptom burden would be among the non-responders, and thus may bias these results.

In the SECA study, the vast majority of patients developed recurrence within 3 years following LT. Despite this, several patients had long OS after relapse partially due to vigorous treatment. There has been some concern whether these patients had an acceptable QoL and enjoyed the years of life gained [43]. In this report, we showed that there was no clinical or statistically significant difference in GHS score from baseline to the last HRQoL follow-up at 3 years for the 14 living patients with recurrence within 3 years, implying a stable QoL despite further surgical and/or oncological treatments. Furthermore, there was not a clinically relevant fall in GHS mean values from the time point prior to relapse to the first time point after relapse in all patients with relapse within 36 months (*n* = 21), suggesting that HRQoL remained stable also in the first months after recurrence.

It seems that the SECA patients had more diarrhea compared to the NORDIC-VII patients and the control population, and this symptom tended to continue for several years after LT (Table 3) [33]. Despite the increased rate of diarrhea, the patients maintained a stable GHS, which may partially be explained by the response shift, in which patients psychologically adapt to their changing health status over time [44]. This phenomenon is well known in various clinical settings, but it may be particularly true for the SECA patients who were given the chance of long-term survival after LT relative to the predicted median OS of 6 months to about 2 years if they had not received LT. These results are consistent with our previous study where we reported higher incidence of diarrhea grade 3–4 in transplanted patients receiving palliative chemotherapy after LT compared to prior to LT [45]. Following solid organ transplantation, diarrhea is often observed and is a source of significant morbidity and occasional mortality [46-48]. The two most common causes for diarrhea after LT are infection and side effects of immunosuppressant medications [48]. The incidence of diarrhea seems to be higher with tacrolimus than cyclosporine A [49, 50]. In patients treated with the mTOR inhibitor sirolimus, however, diarrhea seems to be less frequent compared to other immunosuppressants [34, 51], although the incidence appears to increase with higher doses of sirolimus [52, 53]. In the SECA study, all patients were treated with mycophenolate mofetil and a relatively high dose of sirolimus [6], which may explain the increased incidence of diarrhea in these patients.

Some limitations in this report should be noted. This is not a randomized study, but a retrospective comparison of prospective collected data from two different study cohorts. However, we aimed to compare the SECA cohort to patients with similar characteristics (metastatic site, age, sex, KRAS/BRAF status, and ECOG status) from the NORDIC-VII study (Table 1). Despite a relapse in most of the LT patients, the GHS remained good.

## Data Availability

The raw data supporting the conclusion of this article will be made available by the authors, without undue reservation.

## Abbreviations

CRC, colorectal cancer; CRLM, colorectal cancer liver metastases; CRP, C-reactive protein; ECOG, Eastern Cooperative Oncology Group; GHS, Global health score; HRQoL, health-related quality of life; LT, liver transplantation; mCRC, metastatic colorectal cancer; OS, overall survival; PET, positron emission topography; PD, progressive disease; PF, physical function; QoL, quality of life; TUDD, time until definitive deterioration.
