# Supplementary material for: Health-Related Quality of Life in Colorectal Cancer Patients Treated With Liver Transplantation Compared to Chemotherapy
Source: Transpl Int. 2022 May 30;35:10404. doi: 10.3389/ti.2022.10404 (PMC9189292; doi:10.3389/ti.2022.10404)
Supplement: Supplementary file 1 [file DataSheet1.pdf]

Supplementary Table S1: Chemotherapy prior to liver transplantation

Number of lines n (%)	
**One line**	10 (43)
**Two lines**	9 (39)
**Three lines**	4 (18)
Chemotherapy received, n	
**5-FU**	23
**Irinotecan**	18
**Oxaliplatin**	19
**Bevazicumab**	9
**Cetuximab**	5
**Preoperative chemoradiotherapy**	3

Supplementary Table S2: Baseline HRQoL mean values. One line of chemotherapy compared to two or three lines of chemotherapy prior to LT in the SECA cohort.

	1 line<br>n=10	2-3 lines<br>n=13	P-value[1]
**Physical Functioning**	87.8	90.2	0.410
**Social Functioning**	65.0	75.6	0.522
**Role Functioning**	80.0	83.4	0.784
**Emotional Functioning**	76.7	85.9	0.313
**Cognitive Functioning**	88.3	87.2	0.522
**Global QOL**	75.0	78.8	0.784
**Fatigue**	25.6	28.2	0.693
**Nausea/Vomiting**	5.0	3.8	0.976
**Pain**	8.3	14.1	0.648
**Dyspnoea**	6.7	7.7	0.927
**Sleeping Disturbances**	23.3	28.2	0.693
**Appetite Loss**	6.7	10.2	0.483
**Constipation**	20.0	7.7	0.208
**Diarrhoea**	13.4	15.4	0.832
**Financial Impact**	3.4	28.2	0.208

[1] Mann-Whitney U test.

Abbreviations: QOL, Quality of life.

Supplementary Table S3: Baseline mean values in patients with progressive disease before and after six months in the N-VII-310 and the N-VII-43 cohorts.

	PFS<6 months (N-VII-310)	PFS>6 months (N-VII-310)	P-value[1]	PFS<6 months (N-VII-43)	PFS>6 months (N-VII-43)	P-value[2]
**Physical Functioning**	82.4	86.7	0.117	80.8	88.0	0.544
**Social Functioning**	77.9	81.5	0.239	78.9	85.7	0.381
**Role Functioning**	65.2	72.7	0.067	55.5	76.2	0.059
**Emotional Functioning**	73.1	77.2	0.104	75.5	82.7	0.485
**Cognitive Functioning**	87.9	90.6	0.136	92.2	95.8	0.430
**Global QOL**	65.6	70.5	0.056	63.8	72.9	0.259
**Fatigue**	34.3	29.6	0.096	35.5	25.8	0.129
**Nausea/Vomiting**	7.4	6.4	0.214	4.44	7.14	0.666
**Pain**	22.0	20.5	0.624	21.1	14.3	0.631
**Dyspnoea**	15.8	13.7	0.334	13.3	10.7	0.452
**Sleeping Disturbances**	32.3	23.2	0.008	20.0	17.8	0.920
**Appetite Loss**	22.6	18.6	0.198	22.2	16.6	0.462
**Constipation**	13.6	14.0	0.931	24.4	5.9	0.012
**Diarrhoea**	14.5	15.6	0.945	17.8	13.5	0.487
**Financial Impact**	12.5	9.7	0.160	17.8	10.7	0.105

[1] N-VII-310 cohort, Mann-Whitney U test. [2]N-VII-43 cohort, Mann-Whitney U test

Abbreviations: N-VII-43, NORDIC-VII liver-only cohort, 43 patients; N-VII-310, NORDIC-VII cohort (including patients with extra-hepatic metastatic disease), 310 patients; QOL, Quality of life
